# Anorectal Malformations and Hirschsprung Disease: A 30-Year Retrospective Outreach Review

**DOI:** 10.7759/cureus.79987

**Published:** 2025-03-03

**Authors:** Patrick A Dewan, Patrick J Mill

**Affiliations:** 1 Paediatric Surgery, Sunshine Private Hospital, Melbourne, AUS; 2 Paediatrics, Oceania University of Medicine, Apia, WSM

**Keywords:** anorectal malformations, congenital anomalies, functional bowel outcomes, hirschsprung disease, pediatric surgery, posterior sagittal anorectoplasty, postoperative complications, reoperative procedures, stricture formation, surgical outcomes

## Abstract

Objective

This study analyses the indications for and outcomes of primary and redo surgeries for anorectal malformations (ARMs) and Hirschsprung disease (HD) in developing, resource-limited countries. The study seeks to identify trends in primary surgery complication rates, evaluate the indications for reoperation and explore potential strategies to improve surgical management and long-term outcomes for children with ARM and HD in these settings.

Methodology

A retrospective cohort analysis was conducted on data collected by the Kind Cut for Kids (KCFK) surgical outreach program, a charitable initiative that provides paediatric surgical care in under-served regions. Data was collected over a 30-year period across 22 developing countries. The dataset contained 2,498 observations linked to ARM or HD, which was filtered to include those with primary surgeries, reoperations for prior complications and management of complications of any surgery done by the visiting team. Clinical data included demographics, pathology classification, surgical details, and postoperative outcomes.

Results

The final cohort included 496 ARM patients and 224 HD patients, with 65% and 41%, respectively, undergoing primary corrective surgeries. Among ARM cases, 25% required redo surgeries, with malposition (33%), strictures (24%), or prolapse (8%) being the most common indications. In HD, 45% of patients required redo procedures, primarily for strictures (19%), prolapse (9%), or acquired fistulas (4%).

The most common redo procedures for ARMs were the posterior sagittal anorectoplasty (PSARP) (58%) or anorectal angle plication (10%). For HD patients, PSARP (11%) and the Swenson procedure (10%) were the most common corrective redo procedures. Due to the focus of KCFK visits, there are significant data gaps pertaining to primary surgical details, reoperation indications and follow-up data. This reflects the challenges of managing these conditions in resource-limited environments and with an outreach program.

Conclusion

This study highlights high rates of complications from primary surgeries in ARM and HD cases treated in resource-limited settings, which emphasises the need for enhanced surgical precision, structured postoperative care, and consistent follow-up protocols, as well as education of surgeons in the countries visited. Targeted interventions such as capacity-building initiatives, tailored consensus guidelines, and telemedicine integration are critical to addressing disparities in care. Future prospective studies with standardised data collection and outcome metrics are essential to validate these findings and improve care delivery for children with ARM and HD in underserved regions.

## Introduction

Anorectal malformations (ARMs) are a spectrum of congenital anomalies in which the anus is either absent or abnormally located outside the normal sphincter complex [1]. Cases range from mild malformations such as stenosis to more complex cases involving fistulas connecting the rectum and urogenital structures or cloacal defects, which may involve multiple organ systems [2-4]. Patients present with an absent anal opening and signs of bowel obstruction [5]. Diagnosis is clinical, supported by imaging and screening for VACTERL associations [6].

Early surgical intervention is preferable as it is considered important to ensure ARM cases have the best functional outcome. Posterior sagittal anorectoplasty (PSARP) remains the standard approach for managing ARMs, designed to restore anatomy and function as closely as possible to normal [7,8], though achieving durable outcomes remains challenging, especially in severe cases, particularly where muscle and bone development are limited.

Hirschsprung disease (HD) is a congenital disorder due to the absence of ganglion cells in the bowel, which results in functional bowel obstruction due to a lack of relaxation of the distal bowel [9]. The aganglionic segment most commonly involves the rectosigmoid region but can involve the whole colon in 5% of cases [10]. The disease most usually presents in the neonatal period, with infants suffering from abdominal distention, bilious vomiting and failure to pass meconium [11], although mild cases can go undiagnosed until later in infancy and childhood [12]. The definitive diagnosis is confirmed with rectal biopsy, which demonstrates absent ganglion cells [13]. The treatment of choice is surgical resection and connection of a ganglionic segment to the anal canal [14]. Despite advances in surgical techniques such as the Swenson, Soave, and Duhamel procedures, postoperative complications remain a significant concern [15].

Functional outcomes following ARM and HD surgeries are generally favourable [16]. However, a subset of patients develop complications that necessitate reoperative procedures [17,18]. These complications can include anal malposition, stricture, and prolapse, as well as constipation and incontinence [19]. Complication rates from primary ARM and HD surgeries vary widely in the literature, with reoperation rates ranging from 15 to 40% for ARMs [7,8] and 20 to 40% for HD [20,21]. These rates are influenced by the surgical technique, patient population and follow-up duration considerations. Additionally, reoperation rates from studies in high-resource settings are reported at 10-20%, whilst being significantly higher in developing countries, with rates up to 50% [22]. These inconsistencies are further compounded by the lack of standardized reporting metrics [23], which makes the comparison of outcomes across studies difficult.

Our study hypothesises that high complication and reoperation rates following primary ARM and HD surgeries are influenced by variations in surgical techniques, patient populations and follow-up practices. Utilising data collected over 30 years from the KCFK program, which operates only in developing countries, our study evaluates a large, geographically diverse cohort to identify trends in complications and surgical outcomes leading to reoperation. These findings aim to highlight areas that can be developed to improve the management of patients and enhance the long-term outcomes for children affected by ARM and HD in these regions.

## Materials and methods

Study design and cohort identification 

This retrospective cohort study analysed data collected by the Kind Cuts for Kids (KCFK) program, which has been providing specialized surgical care to children with complex congenital anomalies in resource-limited settings for more than three decades. Between 1993 and 2023, the KCFK program conducted missions in 22 developing countries, delivering surgical interventions and mentoring and training local healthcare providers in advanced surgical techniques to build capacity and improve long-term care. Data fields included patient demographics of age, gender and location, while clinical parameters included consultation types and dates, diagnosed pathology, classification and primary surgical procedures, which included the type of surgery and surgical approach. 

The complete dataset for the KCFK outreach program comprised 9,509 patient observations, representing 3,258 individual patients who underwent a total of 3,871 surgical procedures. Of these, 2,498 observations were associated with a primary diagnosis of ARMs or HD. Geographic distribution of cases was heavily weighted to countries visited most frequently as follows: PNG 925 (37%), Mauritius 694 (28%), Kosovo 382 (15%) and Bosnia 133 (5%). Together, these countries contributed 85% of the total observed cases.

Patient selection and data filtering 

ARM and HD patient records were initially filtered to identify those who had undergone a primary corrective surgery. We have defined primary surgery as any initial corrective surgical procedure performed with the intent to address the anatomical and functional anomalies associated with ARM or HD, regardless of whether the surgery was conducted by the KCFK team or a local provider. This population was then refined to those that had identified postoperative functional or anatomical complications during either the initial postoperative assessment or during representation at a subsequent KCFK visit. These patients were further sorted to identify those who underwent a subsequent secondary surgery.

The final cohort comprised 496 ARM patients and 224 HD patients. Where data was available, analysis was conducted to categorise the types of primary surgeries performed, the complications encountered and the specific indications for reoperation. Where possible, ARM cases were classified according to the Krickenbeck classification system, which categorises ARM based on the presence and type of associated fistulas and the level of the rectal pouch. HD cases were classified based on the length of the aganglionic segment. Where possible, postoperative outcomes were recorded. Anatomical complications included malposition of the anus, strictures, prolapse, and fistula formation. Functional complications were assessed based on symptoms of incontinence, constipation, and overall bowel function. Data on the timing and type of reoperative procedures, along with postoperative follow-up, were also collected. 

For context, KCFK data was not originally collected for statistical or research purposes but for record maintenance as part of informal recording of diagnosis and management for informing donors. As such, there are some gaps in available data, with limited options to more comprehensively populate missing fields. KCFK delivers services via short-duration visits, in coordination with local providers, for complex gastrointestinal and genitourinary paediatric cases. There are often years between return visits, and many patients travel from isolated locations to receive treatment and frequently do not return for follow-up. These factors, combined with the reliance on local providers for initial diagnosis and primary management, as well as the KCFK intervention postoperative management, result in some significant data gaps. These are principally associated with preoperative classification, confirmation of primary surgical procedures and postoperative follow-up and management. 

Statistical analysis 

Descriptive statistics were used to summarise data fields, including patient demographics and clinical data. Analysis of categorical variables was documented as percentages and frequencies, and continuous variables were recorded using medians and interquartile ranges (IQR). All statistical analyses were performed using Stata version 18 (StataCorp LLC, College Station, Texas, USA).

## Results

Of 3258 patients contained in the KCFK database, 496 (15%) were diagnosed with ARM and 224 (7%) with HD. To simplify the distribution of results, details of ARM patients will be presented first, followed by HD. 

ARM cohort analysis

Among the 496 ARM patients, 323 (65%) had previously undergone a primary corrective operation. These operations included 197 (61%) performed by local providers and 126 (39%) by KCFK. The most common procedures were PSARP in 179 patients (55%) and cutback anoplasty in 26 patients (8%). Of these, 46 required an additional laparotomy, and 145 underwent a combined procedure, which included colostomy revision, cystoscopy, or examination under anaesthesia. The median age at primary operation was 6.4 years (IQR 4.2-10.1 years), comprising 186 male patients and 137 female patients. The most frequently recorded ARM subtypes in those who underwent primary surgery were ARM high in 42 (13%) patients, ARM low in 21 (7%), rectovestibular fistula in 19 (6%), and cloaca in 17 (5%). Detailed classification information was not available for 168 (52%) patients, predominately those who had undergone primary procedures by the local surgeons prior to the outreach visit. 

Of the 323 patients who underwent their initial surgery, 242 were noted to have an anatomically correct repair at postoperative review or did not require further intervention. Eighty-one (25%) patients required a redo procedure due to complications from the primary operation, with six of these patients undergoing multiple redo procedures. Redo operations were performed at a median age of 5.9 years and included 46 male patients and 35 female patients. The most common ARM subtypes in patients undergoing redo surgery were ARM high in 12 (15%) cases and cloaca in 18 (22%) cases. Classification details were not available for 49 (61%) of cases. 

In terms of primary operations for those undergoing redo procedures, 12 (15%) had a PSARP, six (7%) had a PSARP with plication, five (6%) had a PSARP with anterior plication, three (4%) had PSARP with perineal body reconstruction, five (6%) had PSARP with posterior plication, and five (6%) underwent rectal resection. The primary operation was not recorded for 45 (56%) patients. 

Indications for redo surgery included malposition in 29 cases (33%), stricture in 21 cases (24%), prolapse in seven cases (8%), and fistula in five cases (6%). For 25 operations (28%), specific indications were not documented. Among the 29 patients requiring redo surgery for malposition, the surgical approach involved PSARP in 15 cases (52%), anorectal angle plication in five cases (17%), anoplasty in two cases (7%), and colostomy revision only in seven cases (24%). For patients with stricture, 15 cases (71%) were managed with PSARP, two cases (10%) with anorectal angle plication, and four cases (19%) required a colostomy revision. In the fistula group, three patients (60%) underwent PSARP, while anorectal angle plication and colostomy revision were used in one case each (20%). For the 25 cases without documented specific indications, the surgical approaches included PSARP in 18 cases (75%), anorectal angle plication in three cases (8%), anoplasty in one case (4%), and colostomy revision in three cases (13%). 

Due to the unique mission and operating conditions of KCFK clinics and variability in post-operative management, comprehensive follow-up data on reoperation outcomes is limited. However, follow-up after three months was recorded for 52 (60%) cases at a median duration of 2.4 years. Summary details are displayed in Table 1. 

**Table 1:** Summary of the Anorectal Malformation (ARM) Cohort PSARP: Posterior sagittal anorectoplasty

Parameter	Value
Total ARM patients	
Patients undergoing primary surgery	323 (65%)
Most common primary surgery (PSARP)	179 (55%)
Median age at primary surgery	6.4 years
Reoperations	81 (25%)
Malposition of anus	29 cases (9%)
Strictures	21 cases (7%)
Prolapse	7 cases (2%)
Median age at reoperation	5.9 years
Most common reoperation	PSARP (58%)

HD cohort analysis 

Of the 224 HD patients, 105 (47%) underwent a primary corrective operation. These operations included 66 (63%) performed by local providers and 39 (37%) by KCFK. The median age at primary surgery was 4.9 years (IQR 3.0-7.5 years), with 79 (75%) male patients and 26 (25%) female patients. Data on the subtype of HD (related to the location of the aganglionic segment) was largely unavailable, with no specific classification recorded for 92 (88%) patients. Surgical approaches for primary correction included the Swenson procedure in 82 patients (78%), of which nine (9%) were single-stage operations. The Soave procedure was documented for 13 (12%) patients, and rectal resection in 10 (10%) patients. In 48 (46%) patients, the primary operation was combined with a colostomy closure, with 38 (79%) of these patients undergoing a Swenson procedure. Multiple primary operations were recorded in eight patients (8%), with rectal resection and the Swenson procedure being the most common combination for six (75%) of these cases. 

Among the patients who underwent primary surgery, 57 (54.3%) either did not require long-term follow-up, had an anatomically correct repair, or were managed locally. Forty-seven (44.7%) patients required redo procedures. Three of these patients had multiple redo operations. Redo surgeries were performed at a median age of 6.5 years, with 28 male patients (60%) and 19 female patients (40%). Redo procedures were secondary to primary Soave procedures in six (13%) patients, Swenson in five patients (11%), PSARP in three (6%) patients and Duhamel in one patient (2%). The primary operation was not documented for 35 (74%) patients. 

Indications for redo procedures included a stricture in nine patients (19%), prolapse in four patients (9%), and acquired fistula in two patients (4%). The indications for redo surgery were not detailed for 35 patients (74%). For stricture cases, surgical management included colostomy revision in seven patients (78%), PSARP in one patient (11%), and Swenson procedure in one patient (11%). Acquired fistula cases were treated with one PSARP (50%) and one colostomy revision (50%). For the 35 patients without specific indications, the surgical procedures were as follows: 10 anorectal angle plication procedures (29%), seven PSARP procedures (20%), five Swenson procedures (14%), one anoplasty (3%) and 12 colostomy revisions (34%).

Follow-up for redo procedures was recorded in 28 (60%) patients, with a median duration of 2.3 years. Summary details are contained in Table 2. 

**Table 2:** Summary of the Hirschsprung Disease (HD) Cohort PSARP: Posterior sagittal anorectoplasty

Parameter	Value
Total HD patients	224
Patients undergoing primary surgery	105 (47%)
Most common primary surgery (Swenson)	82 (78%)
Median age at primary surgery	4.9 years (IQR 3.0–7.5 years)
Reoperations	47 cases (44.7%)
Anastomotic strictures	9 cases (19%)
Prolapse	4 cases (9%)
Median age at reoperation	6.5 years
Most common reoperation	PSARP (11%), Swenson (10%)

## Discussion

This study provides an analysis of outcomes following primary and redo surgeries for patients with ARM and HD. By examining a large cohort with data collected over 30 years in 22 countries, this study is able to identify key trends in primary surgical complications, highlight the need for reoperations, and underscore the challenges associated with delivering care in resource-limited settings. The findings illustrate the complexity of achieving long-term success in these conditions, even with well-established surgical techniques.

Key surgical outcomes

The high rates of redo surgeries observed in both ARM (25%) and HD (45%) cohorts highlight significant challenges in addressing these congenital anomalies [24,25]. These rates reflect the difficulty in achieving both anatomical and functional correction during primary surgery, and the outcomes are more complicated in this study due to primary operations being conducted in resource-limited settings without advanced diagnostic, surgical and post-operative support systems to maximise outcomes. In addition, it should be noted that this cohort is unique in that only severe/complex cases were likely to present within the KCFK framework, suggesting a higher complication rate from the initial surgery was likely. It is expected that milder complications such as stricture requiring dilation were managed locally and therefore not reflected in the dataset. Also, many of the procedures were undertaken by surgeons not trained in Paediatric Surgery.

For ARM patients, the primary complications leading to reoperation, malposition, strictures, and prolapse suggest that while the initial surgeries aimed to restore anatomical alignment, achieving durable functional outcomes remains a challenge. Malposition complications, in particular, highlight the complexity of recreating the anorectal anatomy in a manner that aligns appropriately with the sphincter complex. This typically occurs when the primary anoplasty is not centred in either the anterior-posterior or left right plan and prevents the external sphincter complex from closing [26]. Reoperation requires careful dissection and mobilisation of the rectum to enable repositioning within the centre of the sphincter complex. Malposition is consistently recognised as the most common complication requiring a redo procedure in the literature [27]. Unfortunately, specific malposition classification details are not available in the dataset for more detailed analysis. 

In HD, the prevalence of strictures as a complication emphasises the technical difficulty of achieving a tension-free anastomosis and ensuring adequate blood supply to the remaining bowel [28]. Strictures can present as either superficial skin level strictures or deeper strictures that extend proximally. Superficial strictures can often be managed through outpatient procedures such as the Heineke-Mikulicz stricturoplasty; however, deeper strictures require more extensive procedures that require pulling through healthy bowel to rectify the obstruction [29]. There was insufficient data regarding functional outcomes, including constipation and faecal incontinence, for direct assessment in this study, but they remain major concerns in HD cases. These complications are likely to significantly impact the quality of life of patients and their families, underlining the importance of identifying patients at higher risk of poor outcomes to guide both surgical planning and long-term management strategies.

Surgical approaches and complications

Surgical approaches in this study were consistent with globally recognised best practice techniques. In ARM cases, the PSARP was the predominant procedure for both primary and redo surgeries [30]. This operation was introduced by Peña and DeVries in 1982 and has become the gold standard due to its ability to provide both direct visualization and precise anatomical reconstruction [31]. However, the variability in outcomes seen in this cohort reflects the significant skill and experience that are required to perform this procedure effectively and without complications that require reoperation. The findings also highlight the role of adjunct procedures, such as colostomy revisions and anorectal angle plication, in addressing specific complications like malposition and strictures.

For HD patients, the Swenson procedure was the most commonly performed operation, accounting for 83 (79%) of the primary surgeries, including nine (9%) conducted as single-stage procedures. This approach involves the complete resection of the aganglionic segment and direct anastomosis of the ganglionic bowel to the anus [32]. Despite its recognised effectiveness, multiple studies suggest that the Swenson procedure does increase the risk of stricture complications at the anastomotic site [33]. Although this study lacks the data to confirm the indication for reoperation for 35 (74%) HD cases, stricture was the leading cause of reoperation in 60% of those patients with complete histories. This emphasises the need for surgical precision, particularly to reduce tension and ensure adequate perfusion to the bowel ends. The high complication rates necessitating reoperation (54%) may have been influenced in this cohort by a variety of factors, including resource constraints and variations in surgical expertise.

The Soave procedure, performed in 13 (12%) of primary HD cases, represents an alternative approach that preserves the muscular cuff of the rectum while performing a submucosal resection of the aganglionic bowel. The advantages of this technique are that it reduces the risk of damage to the pelvic nerves and is favoured in cases with long-segment or total colonic aganglionosis [34]. The disadvantages are that the retained muscular cuff increases the potential for stricture formation and the development of obstructive symptoms [35]. While specific data on complications directly related to the Soave technique are limited in this study, 46% of primary Soave procedures resulted in complications requiring a reoperation. As with the Swenson procedure, this suggests that the Soave procedure requires surgical precision and comprehensive postoperative care to achieve long-term surgical-free interventions. Combined procedures, such as colostomy closures, were frequent in this cohort, occurring in 48 (46%) of cases. This demonstrates the staged approach that is often required for the management of HD, especially in resource-limited settings. 

Geographic disparities in outcomes

The geographic distribution of this cohort was heavily weighted towards countries most frequently visited by the KCFK program, with Papua New Guinea, Mauritius, Kosovo and Bosnia representing 85% of data from the total of 22 countries. Consistently, however, these countries represent areas with limited access to specialised paediatric gastrointestinal and genitourinary surgical care, and as such, congenital anomalies like ARM and HD have the potential to remain undiagnosed beyond those timeframes observed in developed countries. The data reveals this in the comparatively high patient age for both primary surgeries and reoperations; surgery before 12 months of age would be expected in countries with more comprehensive dedicated resources [7]. Additionally, while the geographic diversity provides a broad perspective, it also introduces variability in surgical technique and postoperative care, which can impact outcomes. Therefore, the high complication and reoperation rates in these settings are likely multifactorial and influenced by the delay in initial diagnosis, lack of advanced imaging and diagnostic tools, limited specialist surgical expertise and deficient long-term postoperative management systems. 

Data limitations

This study has several data limitations that should be acknowledged. Firstly, there is incomplete documentation of primary surgeries and reoperation indications. In total, 56% of ARM patients lacked complete details of their primary surgery, and specific classification details were absent for 52%. In the HD cohort, 74% of patients had no primary surgery record or detailed indications for their redo procedure. This limits our ability to draw more detailed conclusions about the causes of the complications, the selection of the surgical interventions and the outcomes of their management. Additionally, follow-up data was limited to observation status only for 60% and lacked defined metrics to determine post-operative status and long-term quality of life. 

This study’s retrospective design introduces inherent limitations, including the potential for selection bias and incomplete data fields. The absence of consistent standardised reporting metrics and lack of long-term follow-up data also limit options to make generalisations across the diverse and geographically dispersed patient cohort. Future prospective studies should aim to utilise standardised data collection methods tailored to the unique challenges of studying ARM and HD in resource-limited settings. These methods should seek to consistently record detailed pathology data such as the Krickenbeck classification for ARM and documentation of the length and location of the aganglionic segment in HD [36]. Future studies would also benefit from more comprehensive perioperative data, including diagnostic and imaging results (such as contrast studies, biopsy results and pelvic ultrasounds) and surgical details (including indications, approach, type of repair and intraoperative complications). This would enable results to be compared and generalised more easily across different geographic regions and enable more comprehensive analysis. 

Prospective studies specific to ARM and HD should also aim to standardise postoperative data collection. These should include functional outcome metrics such as continence scores, stool frequency, and quality of life assessments to provide consistent and quantifiable metrics for patient outcomes [37]. Validated tools like the Paediatric Quality of Life Inventory (PedsQL) or bowel function scoring systems would be appropriate. Follow-up intervals should also be defined in the study methodology, with data collection occurring at defined time points, such as six months, one year and three years post surgery. This would enable patient tracking across short, intermediate and long-term outcomes and enable enduring observations for potential complications. 

While these measures are well-established protocols for prospective studies in developed regions, their application within the diverse and resource-limited settings highlighted in this study presents significant challenges. In the short term, digital health solutions such as mobile health platforms and smartphone-based data collection apps could enable real-time recording of patient demographics, diagnostic collection, surgical details and follow-up assessments, even in remote locations. These platforms could be coordinated by local providers without direct input from organisations like KCFK and facilitate the accurate and consistent integration of clinical data into centralised databases across participating locations. Centralised access to data would additionally support telemedicine solutions for complex cases that require specialist care advice not available locally. 

These specific data collection methods could enable future studies to provide a more robust foundation for validating findings, enabling comparisons across diverse populations, and contributing to the development of global best practices for managing ARM and HD.

Implications for clinical practice

The findings of this study emphasise the requirement to establish consensus guidelines for the surgical and postoperative management of ARM and HD in resource-limited settings. While techniques such as PSARP and the Swenson or Soave procedures are recognised globally, the ability of local providers in undeveloped regions to successfully manage these procedures without complication is often compromised by limited access to advanced imaging, specialised tools and surgical training. Solutions such as simulation-based training programs and regional workshops could help improve surgical precision and reduce complications like malposition and strictures, which are significant causes of reoperations in this cohort. 

For HD, the high rate of reoperations following complications from Swenson and Soave procedures highlights the need for practical postoperative monitoring protocols relevant to resource-limited settings. As detailed above, simple follow-up measures such as clinical assessments for obstruction, dietary modification and basic bowel management programs should be emphasised in any consensus guidelines. Incorporating validated functional scoring systems specific for ARM and HD would additionally enable a more comprehensive assessment of patient outcomes whilst remaining feasible to resource-limited centres. By tailoring consensus guidelines to the realities of these countries, healthcare providers can improve surgical outcomes, reduce the need for reoperations, and enhance the quality of life for children with ARM and HD.

The disparities across regions underscore the need for targeted interventions to improve outcomes. Investment in capacity building is essential to sustainably address these issues. Options include training programs designed to upskill local surgeons, infrastructure improvements including imaging and surgical equipment and the development of regional departments with focused ARM and HD care facilities. Perhaps the most sustainable short-term solution is a more comprehensive introduction of telemedicine solutions to leverage international specialist support to provide remote advice and guidance for complex local cases. 

## Conclusions

This study highlights the challenges of managing ARM and HD in resource-limited settings. High rates of complications and reoperations reflect the need for enhanced surgical precision, more comprehensive and structured postoperative care and consistent follow-up protocols. Despite these challenges, the findings highlight the potential for targeted interventions such as capacity-building initiatives, tailored consensus guidelines, and the integration of telemedicine to address disparities in care. The design of this study has also highlighted the need to standardising data collection for future prospective studies to improve data comparability across regions to inform best practice protocols specific to available resources. By prioritizing infrastructure development, education and collaboration, healthcare providers can work toward achieving equitable, high-quality outcomes for children with ARMs and HD in underserved regions.
